# Evaluation of Allplex™ Entero-DR assay for detection of antimicrobial resistance determinants from bacterial cultures

**DOI:** 10.1186/s13104-020-04997-4

**Published:** 2020-03-16

**Authors:** María Fernanda Mojica, Elsa De La Cadena, Adriana Correa, Tobias Manuel Appel, Christian José Pallares, María Virginia Villegas

**Affiliations:** 1 Grupo de Resistencia Antimicrobiana y Epidemiología Hospitalaria, Universidad El Bosque, Ak. 9 #131a-02, Bogotá, DC Colombia; 2 Infectious Diseases Department, School of Medicine, Case Western Reserve University, Cleveland, OH USA; 3 Research Service, Louis Stokes Veterans Affairs Medical Center, Cleveland, OH USA; 4 Universidad Santiago de Cali, Cali, Colombia; 5 Centro Médico Imbanaco, Cali, Colombia

**Keywords:** Multiplex quantitative PCR, Enterobacterales, Enteroccocus spp., Carbapenemases, vanA

## Abstract

**Objective:**

To evaluate the sensitivity and specificity of the Allplex™ Entero-DR, a quantitative PCR-based method, for the detection of β-lactamase-encoding genes and vancomycin-resistance determinants in 156 previously characterized Gram-negative bacilli and *Enterococcus* spp. from bacterial cultures.

**Result:**

The method had 100% sensitivity and between 92 and 100% of specificity for identifying *bla*_KPC_, *bla*_VIM_, *bla*_IMP_, *bla*_NDM_, *bla*_OXA-48-like_, *bla*_CTX-M_ and *vanA*. In nine isolates, unspecific amplifications were detected. The Ct of these false positives was above 33. The Ct of the correctly identified *bla* and *van* genes did not surpass 28 and 30, respectively. None of the clinical isolates included as negative controls yielded any amplification. Therefore, the Allplex™ Entero-DR assay is a highly accurate test for the detection of important antibiotic resistance determinants. With this assay, reliable results can be obtained within 3 h. However, according to our data, samples with Ct values greater than 33 should be considered with caution.

## Introduction

Global dissemination of multi-drug resistant microorganisms is one of the most important public health threats. Infections caused by these organisms are associated with higher mortality and morbidity rates, as well as increased healthcare cost [1]. Moreover, timely administration of appropriate therapy might improve patient outcomes [2]. However, the appropriateness of therapeutic approaches depends not only on phenotypic resistance, but also on the underlying resistance mechanism. Real-time PCR-based assays are able to detect the presence of several genetic resistance determinants regardless of the bacterial species, and are significantly faster compared to phenotypic test, which converts them into valuable screening tools to determine patient’s colonization status and diagnostic tool for clinical decision-making.

Following the US Centers for Disease Control and Prevention, most clinical microbiology laboratories perform culture-based methods, which do not detect the underlying mechanism of carbapenem resistance [3]. Distinguishing carbapenemase producing organisms (CPO) from Gram-negative organisms that are carbapenem resistant due to non-carbapenemase-mediated mechanisms is important, as in most cases, carbapenemase-encoding genes are disseminated via mobile genetic elements (e.g. transposon and/or plasmids) and warrant implementation of more intensive infection control measures [4]. Furthermore, the identification of the specific type of carbapenemase has become imperative to increase the likelihood of therapeutic success and to safeguard the efficacy of new β-lactam–β-lactamase inhibitor combinations, such as ceftazidime–avibactam or meropenem–vaborbactam, which are not active against metallo-β-lactamase (MBL) producers [5].

Currently, there are several commercially available clinical diagnostic options for the detection of carbapenemase-resistant microorganisms. Culture-based methods provide a phenotypic evidence of carbapenem resistance that can be caused by a variety of mechanisms such as carbapenemase production, hyper expression of other β-lactamases, porins mutations or activation of efflux-pumps [4]. Production of carbapenemases can be detected by rapid colorimetric tests (Carba-NP test), the inhibitor-based methods (ethylenediaminetetraacetic acid—EDTA and boronic acid), the carbapenem inactivation method, the modified carbapenem inactivation method and immunochromatographic assays [6]. However, some of them do not discriminate the carbapenemase class present. Furthermore, the co-dissemination of serine and MBL enzymes in the same isolate creates difficulties in their detection [6]. Of special concern are “the big five carbapenemases” (KPC, NDM, VIM, IMP and OXA-48), of which KPC is the most prevalent worldwide [7].

On the other hand, Enterococci are intrinsically resistant to many classes of antibiotics, including β-lactams (penicillins and cephalosporins), aminoglycosides, lincosamides, streptogramins, and trimethoprim-sulfamethoxazole [8]. Consequently, acquisition of additional resistance, such as to vancomycin, makes enterococcal infections very difficult to treat [9]. Although other vancomycin-resistance determinants have been reported, the *vanA* cluster is the most prevalent globally [10]. Additionally, vancomycin resistant Enterococci’s (VRE) capacity to survive for longer periods on inanimate surfaces and its role as a commensal, make its dissemination within health-care facilities difficult to control [9]. Therefore, early identification of resistance genes is important to implement infection control measures and adequate antibiotic therapy, which ultimately impact on the clinical outcome and costs of the health system [11].

The Allplex™ Entero-DR assay (Seegene) is a multiplex qualitative PCR (qPCR)-based test to screen eight resistance genes in Gram-negative bacilli (GNB) and *Enterococcus* spp. Currently, the Allplex™ Entero-DR assay is validated only for diagnostic testing of CPO from rectal swabs [12]. The aim of this work was to evaluate the sensitivity and specificity of the test for the detection of five carbapenemase-encoding genes (*bla*_KPC_, *bla*_NDM_, *bla*_VIM_, *bla*_OXA-48-like_ and *bla*_IMP_), extended-spectrum β-lactamase genes (*bla*_CTX-M_) and vancomycin resistance determinants, *van*A and *van*B, from bacterial cultures, due to the close introduction of the assay in Latin America.

## Main text

### Materials and methods

#### Isolates selection

We used a convenience sample of 156 well-characterized GNB and *Enteroccocus faecium* isolates collected between 2009 and 2019 from Colombian hospitals belonging to an antimicrobial resistance surveillance network. Characterization of these isolates consisted of species ID by automatized methods (Vitek-2 or MALDI-TOF) and detection of antimicrobial resistance determinants by means of an in-house qPCR designed to identify *bla*_CTX-M_, carbapenemase genes (*bla*_KPC_, *bla*_VIM_, *bla*_IMP_, *bla*_NDM_, *bla*_OXA-48-like_), and *vanA* and *vanB* following previously reported conditions [13]. Strains were therefore selected based on their different antibiotic resistance genes. The collection was composed of 118 β-lactamases-producing GNB, 25 *vanA* carrying *E. faecium* isolates and 13 isolates known not to harbor any of the resistance determinants screened (8 GNB and 5 vancomycin-susceptible *E. faecium* isolates). Among the 8 β-lactamase-free GNB included as negative controls, some isolates were resistant to carbapenems by mechanisms other than carbapenemase production (Additional file 1: Table S1). These isolates did not amplify for any of the resistance genes of interest, and tested negative on the Carba-NP assay, confirming the absence of carbapenemases. We also included some previously whole-genome sequenced strains that alongside the results of the qPCR assays were used as a reference to evaluate the Allplex™ Entero-DR assay method.

#### Detection of resistance genes

All procedures were performed according to the Allplex™ Entero-DR protocol, using positive and negative controls provided by the kit in each assembly. Briefly, from frozen stock each isolate was inoculated onto MacConkey agar plates for GNB and BHI agar for enterococci, and incubated for 24 h at 35 °C. Following day, 200 µl of water and 10 µl of Entero-DR IC™ were added to each 1.5 ml tube, which was next inoculated with a single colony taken from a pure culture. After thoroughly mixed, tubes were placed in a thermal block and boiled for 15 min, then centrifuged for 1 min at 15,000×*g* (13,000 rpm) and 5 µl of supernatant was added to the reaction mix of the qPCR. For amplification, we used a LightCycler^®^ CFX96 BioRad (Marnes-la-Coquette, France); results were interpreted by the Seegene System.

The performance of the Allplex™ Entero-DR assay was evaluated in terms of sensitivity, specificity, positive predictive value (PPV) and negative predictive value (NPV), taken the previously obtained results of the in-house qPCR and the whole-genome sequences (WGS) available as gold-standard.

### Results

The complete set of isolates included and the resistance genes each harbored is presented in Additional file 1: Table S1. The majority of the GNB isolates carried *bla*_KPC_ (n = 59), followed by *bla*_CTX-M_ (n = 51) and *bla*_NDM_ (n = 20). Some GNB isolates produced two or three carbapenemases (Additional file 1: Table S1). Due to its low prevalence in Colombia, only two isolates carrying *bla*_OXA-48-like_ and four carrying *bla*_IMP_ were included. Among the 30 *E. faecium* isolates included, 25 isolates harbored *vanA*.

A summary of results is presented in Additional file 2: Table S2. A total of 110 isolates were carbapenemase-producers, 51 harbored *bla*_CTX-M_, 25 were positive for *vanA* and 13 were negative for any antibiotic resistance gene. Noteworthy, the Allplex™ Entero-DR assay did not detect any targeted resistance gene in our negative isolates.

Of the GNB isolates included, 118 were known to carry at least one β-lactamase gene (*bla*_KPC_, *bla*_VIM_, *bla*_NDM_, *bla*_OXA-48-Like_, *bla*_IMP_ and/or *bla*_CTX-M_). As summarized in Additional file 2: Table S2, the majority of these isolates carried *bla*_KPC_, and the most common combination found was *bla*_KPC_ + *bla*_CTX-M_. Notably, some isolates co-carried up to three *bla* genes, as such *bla*_KPC_ + *bla*_CTX-M_ + *bla*_VIM_ and *bla*_KPC_ + *bla*_CTX-M_ + *bla*_NDM_. The sensitivity and specificity values of the test for each targeted gene are shown in Table 1. In general, the sensitivity was 100% for all the screened genes, and the specificity was between 92 and 100%. The assay demonstrated between 100 and 86% PPV and 100% NPV for the targets represented.

**Table 1:** Entero-DR™ Allplex sensitivity and specificity values, calculated by target gene

Genes	% (95% CI)			
	Sensitivity	Specificity	PPV	NPV
*bla*_KPC_	100.0 (99.3–100.0)	100.0 (99.4–100.0)	100.0 (99.3–100.0)	100.0 (99.4–100.0)
*bla*_NDM_	100.0 (98.2–100.0)	99.2 (97.3–100.0)	96.6 (88.2–100.0)	100.0 (99.6–100.0)
*bla*_VIM_	100.0 (97.4–100.0)	99.3 (97.5–100.0)	95.0 (82.9–100.0)	100.0 (99.6–100.0)
*bla*_CTX-M_	100.0 (99.0–100.0)	92.4 (86.8–97.9)	86.4 (76.9–92.02)	100.0 (99.5–100.0)
*vanA*	100.0 (98.0–100.0)	100.0 (99.6–100.0)	100.0 (98.0–100.0)	100.0 (99.6–100.0)

*CI* confidence interval, *PPV* positive predictive value, *NPV* negative predictive value

**Fig. 1:**
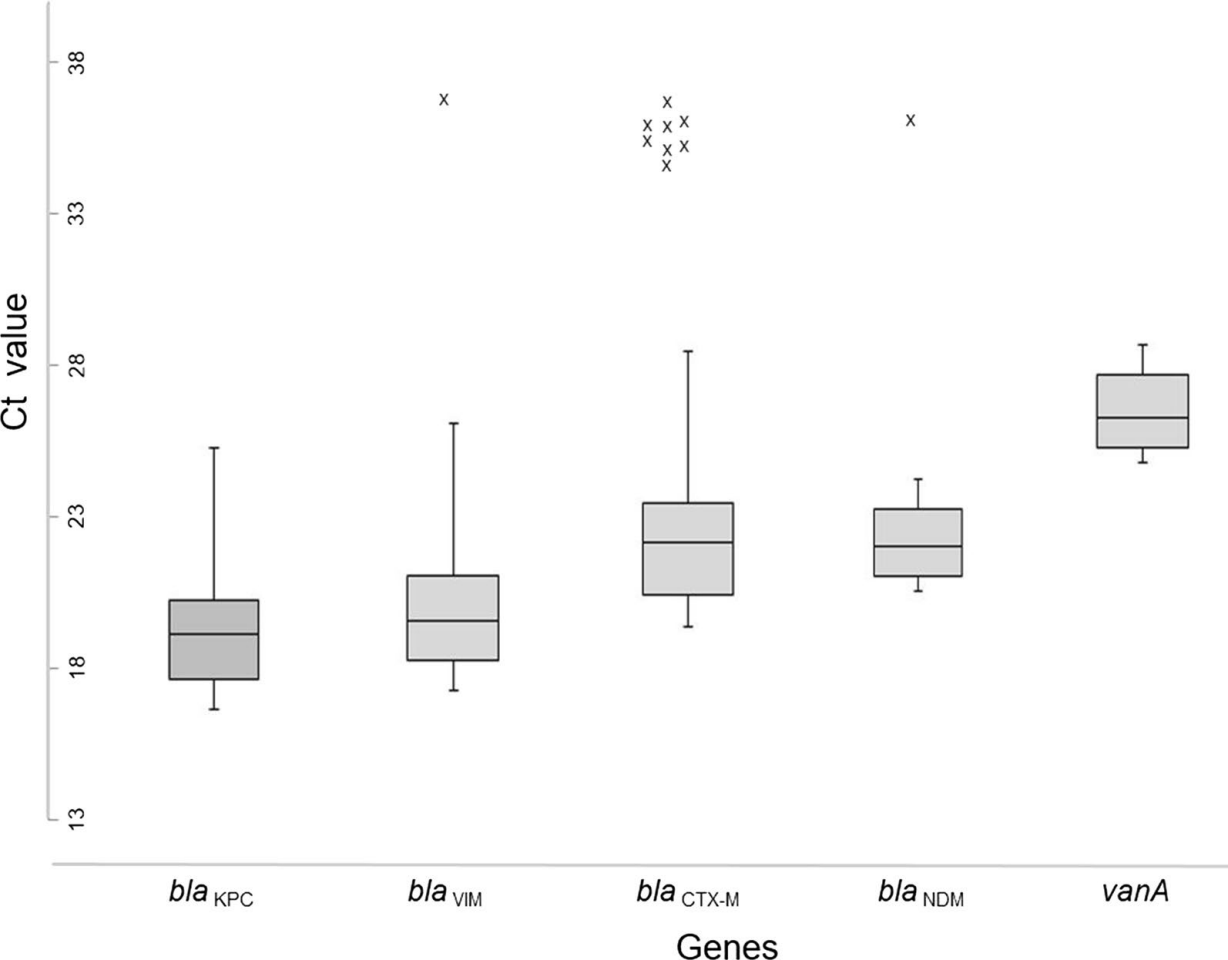
Distribution of the Ct values by antibiotic resistance gene detected. Values marked with (X) are the suspected false positive results

The threshold cycle level (Ct) values for the *bla*_KPC_, *bla*_NDM_, *bla*_VIM_ and *bla*_CTX-M_ genes ranged between 19.4 and 22.5; for *van*A the mean Ct was 26.5 (Fig. 1). The Ct of the correctly identified *bla* and *van* genes did not surpass 28 and 30, respectively. In nine isolates, suspected unspecific amplifications were detected. The Ct of these false positives was above 33 in all cases. None of the clinical isolates included as negative controls yielded any amplification for any targeted gene. The complete set of results of all the isolates tested, including the Ct values of all target genes obtained are shown in Additional file 2: Table S2.

### Discussion

Timely detection of antibiotic resistance determinants such as carbapenemase-encoding genes is necessary not only for the initiation of appropriate antibiotic therapy, but also for the early implementation of infection control measures. Several phenotypic and molecular methods are available. Phenotypic assays are time-consuming, have variable sensitivities toward certain enzymes, and do not identify the exact gene causing the resistance phenotype. Molecular methods, on the other hand, provide a faster and specific diagnosis, but are regarded as more expensive, which can limit their use in low-resource settings [14].

In this work, the performance of Allplex™ Entero-DR, a newly introduced commercial nucleic acid assay test for the detection of the main antibiotic resistant determinants was evaluated. Starting from a pure bacterial culture, the assay provided highly reliable results for 22 samples in 3 h. Comparison with the results previously obtained by means of the in-house qPCR assay and WGS, revealed that all tested isolates carrying resistant genes were correctly identified. The calculated sensitivity and specificity of the assay, 100% and 92–100%, respectively, are in accordance with what has been reported for other commercially available PCR-based assays (Table 2). Notably, the specificity and sensitivity for detecting both *bla*_KPC_ and *bla*_NDM_, the most prevalent carbapenemase-encoding genes found in clinical isolates from Colombia [15, 16], are above 99%. These excellent values, alongside similarly high negative predictive and positive predictive values, foretell an outstanding performance of the Allplex™ Entero-DR assay with this type of samples.

**Table 2:** Comparation of carbapenemase, CTX-M and VanA detecting assays

Test	No	Sens	Spec	Source	Methods	Genes	Turnaround time	References
*Entero-DR assay*	156	100	92–100	Bacterial colonies	Real-time multiplex PCR	*bla*_KPC_, *bla*_VIM_, *bla*_NDM_, *bla*_IMP_ and *bla*_OXA-48-like_	2 h	Present study
KPC	72	100	100					
NDM	28	100	92.2					
VIM	19	100	99.3					
CTX-M	51	100	92.4					
vanA	25	100	100					
IMP	4	(4/4)	99.4					
OXA-48-like	2	(2/2)	100					
	982	100	98.2	Rectal swaps	Real-time multiplex PCR	*bla*_KPC_,* bla*_VIM_, *bla*_NDM_,* bla*_IMP_ and *bla*_OXA-48-like_	2 h	[12]
*Xpert Carba R*	206	95	98.1	Rectal swaps	Real-time multiplex PCR	*bla*_KPC_, *bla*_VIM_, *bla*_NDM_, *bla*_IMP_ and *bla*_OXA-48-like_	1 h	[18]
KPC	120	94.9	99.6					
NDM	61	100	99					
VIM	10	–	99.8					
IMP	9	100	99.8					
OXA-48-like	6	100	99.9					
*CARBA-5 NG*	152	88.2	100		Immunochromatography	*bla*_KPC_, *bla*_VIM_, *bla*_NDM_, *bla*_IMP_ and *bla*_OXA-48-like_	15 min	[19]
KPC	13	100	100					
NDM	29	96.6	100					
VIM	48	100	100					
IMP	9	55.6	100					
OXA-48-like	40	100	100					
*BD-MAX CPO*	175	97.1	98.8	Bacterial colonies and rectal swaps	Real-time multiplex PCR	*bla*_KPC_, *bla*_VIM_, *bla*_NDM_, *bla*_IMP_ and *bla*_OXA-48-like_	1 h	[20]
*BD MAX ESBL screen*	354	95.2	98.8	Rectal swaps	Real-time multiplex PCR	*bla*_CTX-M-1_, *bla*_CTX-M-2_, *bla*_CTX-M-9_ and *bla*_SHV_	1 h	[21]
*Filmarray BCID panel*								
KPC	25	100	100	Blood	Real-time multiplex PCR	*bla*_KPC_, *mecA*, *vanA/B*	1 h	[22, 23]
vanA/B	31	100	100	Blood	Real-time multiplex PCR	*bla*_KPC_, *mecA*, *vanA/B*	1 h	[22, 23]

*No* number of isolates, *Sens* sensitivity, *Spec* specificity

Discrepant results occurred in only 9/156 samples (6%; Fig. 1 and Additional file 2: Table S2). Given that all 9 “false-positives” presented Ct above 33 for the incorrectly detected gene, it is possible that these results could have been caused by sporadic cross-contamination during the reaction set-up process. According to this, positive results with Ct values greater than 33 should be considered with caution, as they may be indicative of false positives. Although no “false negatives” were detected in this study, hypothetically, the heterogeneity within some β-lactamase families (e.g. IMP) could affect the specificity of the primers used in the assay, and hence, could affect diagnostic performance [17].

In conclusion, our results show that the Allplex™ Entero-DR assay is a highly accurate, useful, and fast method, that given its excellent performance, could potentially become an invaluable tool for the early detection of common antibiotic resistance genes among clinical isolates. Since the assay is designed to work with either rectal swabs and from pure bacterial cultures, cost-effectiveness analysis are required to determine the specific need this assay could help mitigate for each health-care institution (e.g. surveillance of resistant bacteria vs. diagnostic tool for therapeutic decisions).

## Limitations

Limitations of this study may be attributed to the low number of positive genes of *bla*_IMP_ and *bla*_OXA-48 like_, and the lack of *vanB* carriers, due to the scarcity of isolates with these genotypes circulating in Colombia.

## Supplementary information

**Additional file 1: Table S1**. Distribution of isolates per resistance determinant(s) harbored, as previously determined by qPCR (n= 156).

**Additional file 2: Table S2**. Complete list of results obtained out of the 156 isolates processed by Allplex ^TM^ Entero-DR assay. Each target amplified with its corresponding Ct are shown. Shadowed values correspond to the presumed false positives results.

## Data Availability

All results are submitted as Additional files.

## Abbreviations

CPO: Carbapenemase-producing organisms
Ct: Threshold cycle level
GNB: Gram-negative bacilli
MBL: Metallo-β-lactamase
NPV: Negative predictive value
PPV: Positive predictive value
VRE: Vancomycin-resistant Enterococci
