# Supplementary material for: Evaluation of Allplex™ Entero-DR assay for detection of antimicrobial resistance determinants from bacterial cultures
Source: BMC Res Notes. 2020 Mar 16;13:154. doi: 10.1186/s13104-020-04997-4 (PMC7075001; doi:10.1186/s13104-020-04997-4)
Supplement: Supplementary file 1 — Additional file 1: Table S1. Distribution of isolates per resistance determinant(s) harbored, as previously determined by qPCR (n= 156). [file 13104_2020_4997_MOESM1_ESM.docx]

|  | **Table S1**. Distribution of isolates per resistance determinant(s) harbored, as previously determined by qPCR (n= 156). | | |
| --- | --- | --- | --- |
|  | Specie | Resistance determinant(s) | No. Of isolates |
|  | *K. pneumoniae* | *bla*_KPC_ | 13 |
|  | *K. pneumoniae* | *bla*_KPC-2_ | 5 |
|  | *K. pneumoniae* | *bla*_KPC-3_ | 11 |
|  | *K. aerogenes* | *bla*_KPC_ | 1 |
|  | *E. cloacae* complex | *bla*_KPC_ | 2 |
|  | *E. cloacae* complex | *bla*_KPC-2_ | 2 |
|  | *S. marcescens* | *bla*_KPC_ | 2 |
|  | *S. marcescens* | *bla*_KPC-2_ | 1 |
|  | *P. aeruginosa* | *bla*_KPC_ | 2 |
|  | *C. freundii* | *bla*_KPC_ - *bla*_VIM_ | 1 |
|  | *P. aeruginosa* | *bla*_KPC_ - *bla*_VIM_ | 2 |
|  | *P. aeruginosa* | *bla*_KPC_ - *bla*_VIM_ - *bla*_CTX-M_ | 2 |
|  | *K. pneumoniae* | *bla*_KPC_ - *bla*_CTX-M_ | 4 |
|  | *C. freundii* | *bla*_KPC_ - *bla*_CTX-M_ | 1 |
|  | *E. cloacae* complex | *bla*_KPC-2_ - *bla*_CTX-M_ | 1 |
|  | *K. pneumoniae* | *bla*_KPC-2_ - *bla*_CTX-M_ | 4 |
|  | *E. cloacae* complex | *bla*_KPC-2_ - *bla*_CTX-M-15_ | 2 |
|  | *S. marcescens* | *bla*_KPC-2_ - *bla*_CTX-M_ | 3 |
|  | *K. pneumoniae* | *bla*_KPC-2_ - *bla*_CTX-M-12_ | 1 |
|  | *K. pneumoniae* | *bla*_KPC-2_ - *bla*_CTX-M-15_ | 1 |
|  | *K. pneumoniae* | *bla*_KPC-3_ - *bla*_CTX-M-15_ | 3 |
|  | *K. pneumoniae* | *bla*_KPC_ - *bla*_NDM_ | 2 |
|  | *K. pneumoniae* | *bla*_KPC_ - *bla*_NDM -_ *bla*_CTX-M_ | 3 |
|  | *E. coli* | *bla*_KPC_ - *bla*_NDM -_ *bla*_CTX-M_ | 1 |
|  | *E. cloacae* complex | *bla*_KPC_ - *bla*_NDM -_ *bla*_VIM_ | 2 |
|  | *E. coli* | *bla*_NDM_ | 1 |
|  | *K. pneumoniae* | *bla*_NDM_ | 4 |
|  | *P. rettgeri* | *bla*_NDM_ | 1 |
|  | *E. coli* | *bla*_NDM-1_ | 1 |
|  | *K. pneumoniae* | *bla*_NDM_ - *bla*_CTX-M_ | 10 |
|  | *S. marcescens* | *bla*_NDM_ - *bla*_CTX-M_ | 1 |
|  | *K. pneumoniae* | *bla*_NDM-1_ - *bla*_CTX-M_ | 1 |
|  | *K. pneumoniae* | *bla*_NDM-1_ - *bla*_CTX-M-15_ | 1 |
|  | *S. marcescens* | *bla*_VIM_ | 1 |
|  | *P. aeruginosa* | *bla*_VIM_ | 1 |
|  | *E. cloacae* complex | *bla*_VIM-2_ | 1 |
|  | *K. aerogenes* | *bla*_VIM-2_ | 1 |
|  | *K. pneumoniae* | *bla*_VIM-23_ | 1 |
|  | *K. pneumoniae* | *bla*_VIM_ - *bla*_CTX-M_ | 1 |
|  | *E. cloacae* complex | *bla*_VIM_ - *bla*_CTX-M_ | 1 |
|  | *P. aeruginosa* | *bla*_VIM_ - *bla*_CTX-M_ | 2 |
|  | *E. coli* | *bla*_VIM_ - *bla*_CTX-M_ | 1 |
|  | *K. pneumoniae* | *bla*_VIM-2_ - *bla*_CTX-M_ | 2 |
|  | *P. aeruginosa* | *bla*_IMP_ | 4 |
|  | *K. pneumoniae* | *bla*_OXA-48_ | 1 |
|  | *K. oxytoca* | *bla_OXA-48_* - *bla*_CTX-M_ | 1 |
|  | *E. coli* | *bla*_CTX-M_ | 6 |
|  | *E. coli* | *bla*_CTX-M-15_ | 2 |
|  | *E. faecium* | *vanA* | 25 |
|  | *K. pneumoniae* | Negative | 1 |
|  | *P. aeruginosa* | Negative | 2 |
|  | *E. coli* | Negative | 3 |
|  | *S. marcescens* | Negative | 2 |
|  | *E. faecium* | Negative | 5 |
|  |  |  |  |
