# Supplementary material for: Evaluation of Allplex™ Entero-DR assay for detection of antimicrobial resistance determinants from bacterial cultures
Source: BMC Res Notes. 2020 Mar 16;13:154. doi: 10.1186/s13104-020-04997-4 (PMC7075001; doi:10.1186/s13104-020-04997-4)
Supplement: Supplementary file 2 — Additional file 2: Table S2. Complete list of results obtained out of the 156 isolates processed by Allplex TM Entero-DR assay. Each target amplified with its corresponding Ct are shown. Shadowed values correspond to the presumed false positives results. [file 13104_2020_4997_MOESM2_ESM.docx]

| **Table S2**. Complete list of results obtained out of the 156 isolates processed by Allplex ^TM^ Entero-DR assay. Each target amplified with its corresponding Ct are shown. Shadowed values correspond to the presumed false positives results. | | | | | | | | | | | | | | | | | | | | | | |
| --- | --- | --- | --- | --- | --- | --- | --- | --- | --- | --- | --- | --- | --- | --- | --- | --- | --- | --- | --- | --- | --- | --- |
| No | Species | *bla*_KPC_ | Ct |  | *bla*_VIM_ | Ct |  | *bla*_NDM_ | Ct |  | *bla*_IMP_ | Ct |  | *bla*_OXA-48-like_ | Ct |  | *bla*_CTX-M_ | Ct |  | *vanA* | Ct |  |
| 1 | kpn | KPC | 22,87 |  |  |  |  |  |  |  |  |  |  |  |  |  |  |  |  |  |  |  |
| 2 | kpn | KPC | 20,67 |  |  |  |  |  |  |  |  |  |  |  |  |  |  |  |  |  |  |  |
| 3 | kpn | KPC | 17,85 |  |  |  |  |  |  |  |  |  |  |  |  |  |  |  |  |  |  |  |
| 4 | kpn | KPC | 19,29 |  |  |  |  |  |  |  |  |  |  |  |  |  |  |  |  |  |  |  |
| 5 | kpn | KPC | 21,43 |  |  |  |  |  |  |  |  |  |  |  |  |  |  |  |  |  |  |  |
| 6 | kpn | KPC | 20,13 |  |  |  |  |  |  |  |  |  |  |  |  |  |  |  |  |  |  |  |
| 7 | kpn | KPC | 21,91 |  |  |  |  |  |  |  |  |  |  |  |  |  |  |  |  |  |  |  |
| 8 | kpn | KPC | 18,5 |  |  |  |  |  |  |  |  |  |  |  |  |  |  |  |  |  |  |  |
| 9 | kpn | KPC | 16,9 |  |  |  |  |  |  |  |  |  |  |  |  |  |  |  |  |  |  |  |
| 10 | kpn | KPC | 16,94 |  |  |  |  |  |  |  |  |  |  |  |  |  |  |  |  |  |  |  |
| 11 | kpn | KPC | 19,35 |  |  |  |  |  |  |  |  |  |  |  |  |  |  |  |  |  |  |  |
| 12 | ecl | KPC | 17,3 |  |  |  |  |  |  |  |  |  |  |  |  |  |  |  |  |  |  |  |
| 13 | ecl | KPC | 16,35 |  |  |  |  |  |  |  |  |  |  |  |  |  |  |  |  |  |  |  |
| 14 | eae | KPC | 17,17 |  |  |  |  |  |  |  |  |  |  |  |  |  |  |  |  |  |  |  |
| 15 | ecl | KPC | 21,85 |  |  |  |  |  |  |  |  |  |  |  |  |  |  |  |  |  |  |  |
| 16 | pae | KPC | 17,83 |  |  |  |  |  |  |  |  |  |  |  |  |  |  |  |  |  |  |  |
| 17 | pae | KPC | 19,64 |  |  |  |  |  |  |  |  |  |  |  |  |  |  |  |  |  |  |  |
| 18 | kpn | KPC | 18,85 |  |  |  |  |  |  |  |  |  |  |  |  |  |  |  |  |  |  |  |
| 19 | kpn | KPC | 19,3 |  |  |  |  |  |  |  |  |  |  |  |  |  |  |  |  |  |  |  |
| 20 | kpn | KPC | 18,06 |  |  |  |  |  |  |  |  |  |  |  |  |  |  |  |  |  |  |  |
| 21 | kpn | KPC | 20,21 |  |  |  |  |  |  |  |  |  |  |  |  |  |  |  |  |  |  |  |
| 22 | sma | KPC | 19,64 |  |  |  |  |  |  |  |  |  |  |  |  |  |  |  |  |  |  |  |
| 23 | kpn | KPC | 22,07 |  |  |  |  |  |  |  |  |  |  |  |  |  |  |  |  |  |  |  |
| 24 | kpn | KPC | 20,48 |  |  |  |  |  |  |  |  |  |  |  |  |  |  |  |  |  |  |  |
| 25 | kpn | KPC | 20,71 |  |  |  |  |  |  |  |  |  |  |  |  |  |  |  |  |  |  |  |
| 26 | kpn | KPC | 21,82 |  |  |  |  |  |  |  |  |  |  |  |  |  |  |  |  |  |  |  |
| 27 | kpn | KPC | 17,15 |  |  |  |  |  |  |  |  |  |  |  |  |  |  |  |  |  |  |  |
| 28 | kpn | KPC | 17,08 |  |  |  |  |  |  |  |  |  |  |  |  |  |  |  |  |  |  |  |
| 29 | kpn | KPC | 16,4 |  |  |  |  |  |  |  |  |  |  |  |  |  |  |  |  |  |  |  |
| 30 | sma | KPC | 18,34 |  |  |  |  |  |  |  |  |  |  |  |  |  |  |  |  |  |  |  |
| 31 | ecl | KPC | 17,23 |  |  |  |  |  |  |  |  |  |  | OXA-48 | 35,21 |  | CTX-M | 33,72 |  |  |  |  |
| 32 | sma | KPC | 24,58 |  |  |  |  |  |  |  |  |  |  |  |  |  |  |  |  |  |  |  |
| 33 | kpn | KPC | 18,72 |  |  |  |  |  |  |  |  |  |  |  |  |  |  |  |  |  |  |  |
| 34 | kpn | KPC | 16,16 |  |  |  |  |  |  |  |  |  |  |  |  |  |  |  |  |  |  |  |
| 35 | kpn | KPC | 18,67 |  |  |  |  |  |  |  |  |  |  |  |  |  |  |  |  |  |  |  |
|  |  |  |  |  |  |  |  |  |  |  |  |  |  |  |  |  |  |  |  |  |  |  |
|  |  |  |  |  |  |  |  |  |  |  |  |  |  |  |  |  |  |  |  |  |  |  |
|  |  |  |  |  |  |  |  |  |  |  |  |  |  |  |  |  |  |  |  |  |  |  |
| No | Species | *bla*_KPC_ | Ct |  | *bla*_VIM_ | Ct |  | *bla*_NDM_ | Ct |  | *bla*_IMP_ | Ct |  | *bla*_OXA-48-like_ | Ct |  | *bla*_CTX-M_ | Ct |  | *vanA* | Ct |  |
| 36 | kpn | KPC | 19,59 |  |  |  |  |  |  |  |  |  |  |  |  |  |  |  |  |  |  |  |
| 37 | kpn | KPC | 19,9 |  |  |  |  |  |  |  |  |  |  |  |  |  |  |  |  |  |  |  |
| 38 | kpn | KPC | 19,26 |  |  |  |  |  |  |  |  |  |  |  |  |  |  |  |  |  |  |  |
| 39 | cfr | KPC | 19,67 |  | VIM | 19,49 |  |  |  |  |  |  |  |  |  |  |  |  |  |  |  |  |
| 40 | pae | KPC | 19,61 |  | VIM | 17,97 |  |  |  |  |  |  |  |  |  |  |  |  |  |  |  |  |
| 41 | pae | KPC | 16,39 |  | VIM | 15,09 |  |  |  |  |  |  |  |  |  |  |  |  |  |  |  |  |
| 42 | pae | KPC | 20,13 |  | VIM | 18,9 |  |  |  |  |  |  |  |  |  |  | CTX-M | 33,64 |  |  |  |  |
| 43 | pae | KPC | 21,19 |  | VIM | 19,92 |  |  |  |  |  |  |  |  |  |  | CTX-M | 33,9 |  |  |  |  |
| 44 | ecl | KPC | 14,77 |  |  |  |  |  |  |  |  |  |  |  |  |  | CTX-M | 17,08 |  |  |  |  |
| 45 | kpn | KPC | 19,66 |  |  |  |  |  |  |  |  |  |  |  |  |  | CTX-M | 24,87 |  |  |  |  |
| 46 | kpn | KPC | 21,52 |  |  |  |  |  |  |  |  |  |  |  |  |  | CTX-M | 23,63 |  |  |  |  |
| 47 | cfr | KPC | 20,34 |  |  |  |  |  |  |  |  |  |  |  |  |  | CTX-M | 22,38 |  |  |  |  |
| 48 | ecl | KPC | 20,4 |  |  |  |  |  |  |  |  |  |  |  |  |  | CTX-M | 24,23 |  |  |  |  |
| 49 | ecl | KPC | 20,43 |  |  |  |  |  |  |  |  |  |  |  |  |  | CTX-M | 20,61 |  |  |  |  |
| 50 | sma | KPC | 20,85 |  |  |  |  |  |  |  |  |  |  |  |  |  | CTX-M | 21,24 |  |  |  |  |
| 51 | kpn | KPC | 22,04 |  |  |  |  |  |  |  |  |  |  |  |  |  | CTX-M | 21,88 |  |  |  |  |
| 52 | kpn | KPC | 20,12 |  |  |  |  |  |  |  |  |  |  |  |  |  | CTX-M | 21,8 |  |  |  |  |
| 53 | kpn | KPC | 17,37 |  |  |  |  |  |  |  |  |  |  |  |  |  | CTX-M | 20,8 |  |  |  |  |
| 54 | kpn | KPC | 18,87 |  |  |  |  |  |  |  |  |  |  |  |  |  | CTX-M | 23,93 |  |  |  |  |
| 55 | kpn | KPC | 17,84 |  |  |  |  |  |  |  |  |  |  |  |  |  | CTX-M | 20,7 |  |  |  |  |
| 56 | sma | KPC | 17,26 |  |  |  |  |  |  |  |  |  |  |  |  |  | CTX-M | 18,02 |  |  |  |  |
| 57 | kpn | KPC | 19,43 |  |  |  |  |  |  |  |  |  |  |  |  |  | CTX-M | 22,81 |  |  |  |  |
| 58 | sma | KPC | 17,95 |  |  |  |  |  |  |  |  |  |  |  |  |  | CTX-M | 18,65 |  |  |  |  |
| 59 | kpn | KPC | 18,27 |  |  |  |  |  |  |  |  |  |  |  |  |  | CTX-M | 23,17 |  |  |  |  |
| 60 | kpn | KPC | 16,59 |  |  |  |  |  |  |  |  |  |  |  |  |  | CTX-M | 19,58 |  |  |  |  |
| 61 | kpn | KPC | 18,67 |  |  |  |  |  |  |  |  |  |  |  |  |  | CTX-M | 23,91 |  |  |  |  |
| 62 | kpn | KPC | 18,14 |  |  |  |  |  |  |  |  |  |  |  |  |  | CTX-M | 23,3 |  |  |  |  |
| 63 | kpn | KPC | 18,51 |  |  |  |  |  |  |  |  |  |  |  |  |  | CTX-M | 21,84 |  |  |  |  |
| 64 | kpn | KPC | 18,76 |  | VIM | 36,09 |  | NDM | 35,97 |  |  |  |  |  |  |  | CTX-M | 20,42 |  |  |  |  |
| 65 | kpn | KPC | 18,84 |  |  |  |  | NDM | 21,7 |  |  |  |  |  |  |  |  |  |  |  |  |  |
| 66 | kpn | KPC | 16,9 |  |  |  |  | NDM | 24,3 |  |  |  |  |  |  |  |  |  |  |  |  |  |
| 67 | kpn | KPC | 21,36 |  |  |  |  | NDM | 26,45 |  |  |  |  |  |  |  | CTX-M | 21.96 |  |  |  |  |
| 68 | kpn | KPC | 19,66 |  |  |  |  | NDM | 24,81 |  |  |  |  |  |  |  | CTX-M | 20,69 |  |  |  |  |
| 69 | eco | KPC | 19,17 |  |  |  |  | NDM | 22,15 |  |  |  |  |  |  |  | CTX-M | 21,49 |  |  |  |  |
| 70 | kpn | KPC | 17,19 |  |  |  |  | NDM | 21,67 |  |  |  |  |  |  |  | CTX-M | 20,76 |  |  |  |  |
|  |  |  |  |  |  |  |  |  |  |  |  |  |  |  |  |  |  |  |  |  |  |  |
|  |  |  |  |  |  |  |  |  |  |  |  |  |  |  |  |  |  |  |  |  |  |  |
|  |  |  |  |  |  |  |  |  |  |  |  |  |  |  |  |  |  |  |  |  |  |  |
| No | Species | *bla*_KPC_ | Ct |  | *bla*_VIM_ | Ct |  | *bla*_NDM_ | Ct |  | *bla*_IMP_ | Ct |  | *bla*_OXA-48-like_ | Ct |  | *bla*_CTX-M_ | Ct |  | *vanA* | Ct |  |
| 71 | ecl | KPC | 21,09 |  | VIM | 20,65 |  | NDM | 25,41 |  |  |  |  |  |  |  | CTX-M | 34,85 |  |  |  |  |
| 72 | ecl | KPC | 21,59 |  | VIM | 21 |  | NDM | 26,4 |  |  |  |  |  |  |  | CTX-M | 34.14 |  |  |  |  |
| 73 | eco |  |  |  |  |  |  | NDM | 23,17 |  |  |  |  |  |  |  |  |  |  |  |  |  |
| 74 | kpn |  |  |  |  |  |  | NDM | 20,96 |  |  |  |  |  |  |  |  |  |  |  |  |  |
| 75 | pvr |  |  |  |  |  |  | NDM | 23,77 |  |  |  |  |  |  |  |  |  |  |  |  |  |
| 76 | kpn |  |  |  |  |  |  | NDM | 21,4 |  |  |  |  |  |  |  |  |  |  |  |  |  |
| 77 | kpn |  |  |  |  |  |  | NDM | 20,86 |  |  |  |  |  |  |  |  |  |  |  |  |  |
| 78 | eco |  |  |  |  |  |  | NDM | 21,68 |  |  |  |  |  |  |  |  |  |  |  |  |  |
| 79 | kpn |  |  |  |  |  |  | NDM | 23,44 |  |  |  |  |  |  |  |  |  |  |  |  |  |
| 80 | sma |  |  |  |  |  |  | NDM | 22,25 |  |  |  |  |  |  |  | CTX-M | 20,23 |  |  |  |  |
| 81 | kpn |  |  |  |  |  |  | NDM | 20,08 |  |  |  |  |  |  |  | CTX-M | 18,59 |  |  |  |  |
| 82 | kpn |  |  |  |  |  |  | NDM | 22,25 |  |  |  |  |  |  |  | CTX-M | 22,05 |  |  |  |  |
| 83 | kpn |  |  |  |  |  |  | NDM | 23,3 |  |  |  |  |  |  |  | CTX-M | 23,58 |  |  |  |  |
| 84 | kpn |  |  |  |  |  |  | NDM | 25 |  |  |  |  |  |  |  | CTX-M | 25,83 |  |  |  |  |
| 85 | kpn |  |  |  |  |  |  | NDM | 22,89 |  |  |  |  |  |  |  | CTX-M | 23,6 |  |  |  |  |
| 86 | kpn |  |  |  |  |  |  | NDM | 19,65 |  |  |  |  |  |  |  | CTX-M | 19,78 |  |  |  |  |
| 87 | kpn |  |  |  |  |  |  | NDM | 22,82 |  |  |  |  |  |  |  | CTX-M | 22,58 |  |  |  |  |
| 88 | kpn |  |  |  |  |  |  | NDM | 21,19 |  |  |  |  |  |  |  | CTX-M | 21,38 |  |  |  |  |
| 89 | kpn |  |  |  |  |  |  | NDM | 22,02 |  |  |  |  |  |  |  | CTX-M | 22,58 |  |  |  |  |
| 90 | kpn |  |  |  |  |  |  | NDM | 23,33 |  |  |  |  |  |  |  | CTX-M | 22,66 |  |  |  |  |
| 91 | kpn |  |  |  |  |  |  | NDM | 20,81 |  |  |  |  |  |  |  | CTX-M | 19,31 |  |  |  |  |
| 92 | kpn |  |  |  |  |  |  | NDM | 17,41 |  |  |  |  |  |  |  | CTX-M | 22,05 |  |  |  |  |
| 93 | ecl |  |  |  | VIM | 18,2 |  |  |  |  |  |  |  |  |  |  |  |  |  |  |  |  |
| 94 | sma |  |  |  | VIM | 17,53 |  |  |  |  |  |  |  |  |  |  |  |  |  |  |  |  |
| 95 | eae |  |  |  | VIM | 21,69 |  |  |  |  |  |  |  |  |  |  |  |  |  |  |  |  |
| 96 | kpn |  |  |  | VIM | 23,61 |  |  |  |  |  |  |  |  |  |  |  |  |  |  |  |  |
| 97 | kpn |  |  |  | VIM | 19 |  |  |  |  |  |  |  |  |  |  | CTX-M | 33,69 |  |  |  |  |
| 98 | pae |  |  |  | VIM | 20,18 |  |  |  |  |  |  |  |  |  |  | CTX-M | 35,51 |  |  |  |  |
| 99 | pae |  |  |  | VIM | 19,32 |  |  |  |  |  |  |  |  |  |  | CTX-M | 35,12 |  |  |  |  |
| 100 | pae |  |  |  | VIM | 18,46 |  |  |  |  |  |  |  |  |  |  |  |  |  |  |  |  |
|  |  |  |  |  |  |  |  |  |  |  |  |  |  |  |  |  |  |  |  |  |  |  |
|  |  |  |  |  |  |  |  |  |  |  |  |  |  |  |  |  |  |  |  |  |  |  |
|  |  |  |  |  |  |  |  |  |  |  |  |  |  |  |  |  |  |  |  |  |  |  |
| No | Species | *bla*_KPC_ | Ct |  | *bla*_VIM_ | Ct |  | *bla*_NDM_ | Ct |  | *bla*_IMP_ | Ct |  | *bla*_OXA-48-like_ | Ct |  | *bla*_CTX-M_ | Ct |  | *vanA* | Ct |  |
| 101 | eco |  |  |  | VIM | 20,96 |  |  |  |  |  |  |  |  |  |  | CTX-M | 25,37 |  |  |  |  |
| 102 | kpn |  |  |  | VIM | 22,15 |  |  |  |  |  |  |  |  |  |  | CTX-M | 27,93 |  |  |  |  |
| 103 | kpn |  |  |  | VIM | 17,89 |  |  |  |  |  |  |  |  |  |  | CTX-M | 23,75 |  |  |  |  |
| 104 | ecl |  |  |  | VIM | 22,48 |  |  |  |  |  |  |  |  |  |  | CTX-M | 25,38 |  |  |  |  |
| 105 | pae |  |  |  |  |  |  |  |  |  | IMP | 13,76 |  |  |  |  |  |  |  |  |  |  |
| 106 | pae |  |  |  |  |  |  |  |  |  | IMP | 20,77 |  |  |  |  |  |  |  |  |  |  |
| 107 | pae |  |  |  |  |  |  |  |  |  | IMP | 23,42 |  |  |  |  |  |  |  |  |  |  |
| 108 | pae |  |  |  |  |  |  |  |  |  | IMP | 17,27 |  |  |  |  |  |  |  |  |  |  |
| 109 | kox |  |  |  |  |  |  |  |  |  |  |  |  | OXA-48 | 20,37 |  |  |  |  |  |  |  |
| 110 | kpn |  |  |  |  |  |  |  |  |  |  |  |  | OXA-48 | 20,63 |  | CTX-M | 22,83 |  |  |  |  |
| 111 | eco |  |  |  |  |  |  |  |  |  |  |  |  |  |  |  | CTX-M | 23 |  |  |  |  |
| 112 | eco |  |  |  |  |  |  |  |  |  |  |  |  |  |  |  | CTX-M | 20,32 |  |  |  |  |
| 113 | eco |  |  |  |  |  |  |  |  |  |  |  |  |  |  |  | CTX-M | 25,06 |  |  |  |  |
| 114 | eco |  |  |  |  |  |  |  |  |  |  |  |  |  |  |  | CTX-M | 22,91 |  |  |  |  |
| 115 | eco |  |  |  |  |  |  |  |  |  |  |  |  |  |  |  | CTX-M | 24,93 |  |  |  |  |
| 116 | eco |  |  |  |  |  |  |  |  |  |  |  |  |  |  |  | CTX-M | 23,85 |  |  |  |  |
| 117 | eco |  |  |  |  |  |  |  |  |  |  |  |  |  |  |  | CTX-M | 23,19 |  |  |  |  |
| 118 | eco |  |  |  |  |  |  |  |  |  |  |  |  |  |  |  | CTX-M | 23,98 |  |  |  |  |
| 119 | efm |  |  |  |  |  |  |  |  |  |  |  |  |  |  |  |  |  |  | vanA | 24,62 |  |
| 120 | efm |  |  |  |  |  |  |  |  |  |  |  |  |  |  |  |  |  |  | vanA | 28,27 |  |
| 121 | efm |  |  |  |  |  |  |  |  |  |  |  |  |  |  |  |  |  |  | vanA | 27 |  |
| 122 | efm |  |  |  |  |  |  |  |  |  |  |  |  |  |  |  |  |  |  | vanA | 28,49 |  |
| 123 | efm |  |  |  |  |  |  |  |  |  |  |  |  |  |  |  |  |  |  | vanA | 27,84 |  |
| 124 | efm |  |  |  |  |  |  |  |  |  |  |  |  |  |  |  |  |  |  | vanA | 26,25 |  |
| 125 | efm |  |  |  |  |  |  |  |  |  |  |  |  |  |  |  |  |  |  | vanA | 26,39 |  |
| 126 | efm |  |  |  |  |  |  |  |  |  |  |  |  |  |  |  |  |  |  | vanA | 26,51 |  |
| 127 | efm |  |  |  |  |  |  |  |  |  |  |  |  |  |  |  |  |  |  | vanA | 23,78 |  |
| 128 | efm |  |  |  |  |  |  |  |  |  |  |  |  |  |  |  |  |  |  | vanA | 26,73 |  |
| 129 | efm |  |  |  |  |  |  |  |  |  |  |  |  |  |  |  |  |  |  | vanA | 25,42 |  |
| 130 | efm |  |  |  |  |  |  |  |  |  |  |  |  |  |  |  |  |  |  | vanA | 28,06 |  |
|  |  |  |  |  |  |  |  |  |  |  |  |  |  |  |  |  |  |  |  |  |  |  |
|  |  |  |  |  |  |  |  |  |  |  |  |  |  |  |  |  |  |  |  |  |  |  |
|  |  |  |  |  |  |  |  |  |  |  |  |  |  |  |  |  |  |  |  |  |  |  |
| No | Species | *bla*_KPC_ | Ct |  | *bla*_VIM_ | Ct |  | *bla*_NDM_ | Ct |  | *bla*_IMP_ | Ct |  | *bla*_OXA-48-like_ | Ct |  | *bla*_CTX-M_ | Ct |  | *vanA* | Ct |  |
| 131 | efm |  |  |  |  |  |  |  |  |  |  |  |  |  |  |  |  |  |  | vanA | 29,06 |  |
| 132 | efm |  |  |  |  |  |  |  |  |  |  |  |  |  |  |  |  |  |  | vanA | 25,61 |  |
| 133 | efm |  |  |  |  |  |  |  |  |  |  |  |  |  |  |  |  |  |  | vanA | 25,25 |  |
| 134 | efm |  |  |  |  |  |  |  |  |  |  |  |  |  |  |  |  |  |  | vanA | 29,16 |  |
| 135 | efm |  |  |  |  |  |  |  |  |  |  |  |  |  |  |  |  |  |  | vanA | 28,95 |  |
| 136 | efm |  |  |  |  |  |  |  |  |  |  |  |  |  |  |  |  |  |  | vanA | 26,72 |  |
| 137 | efm |  |  |  |  |  |  |  |  |  |  |  |  |  |  |  |  |  |  | vanA | 25,48 |  |
| 138 | efm |  |  |  |  |  |  |  |  |  |  |  |  |  |  |  |  |  |  | vanA | 27,22 |  |
| 139 | efm |  |  |  |  |  |  |  |  |  |  |  |  |  |  |  |  |  |  | vanA | 25,82 |  |
| 140 | efm |  |  |  |  |  |  |  |  |  |  |  |  |  |  |  |  |  |  | vanA | 26,88 |  |
| 141 | efm |  |  |  |  |  |  |  |  |  |  |  |  |  |  |  |  |  |  | vanA | 25,59 |  |
| 142 | efm |  |  |  |  |  |  |  |  |  |  |  |  |  |  |  |  |  |  | vanA | 26,1 |  |
| 143 | efm |  |  |  |  |  |  |  |  |  |  |  |  |  |  |  |  |  |  | vanA | 24,47 |  |
|  |  |  |  |  |  |  |  |  |  |  |  |  |  |  |  |  |  |  |  |  |  |  |

Species: cfr – *Citrobacter freundii*; eae – *Klebsiella aerogenes*; eco – *Escherichia coli*; ecl – *Enterobacter cloacae*; efm – *Enterococcus faecium*; kpn – *K. pneumoniae*; kox – *K. oxytoca*; pae – *Pseudomonas aeruginosa*; sma – *Serratia marcescens*;
